# Rice (*Oryza sativa* L.) cytochrome P450 protein 716A subfamily CYP716A16 regulates disease resistance

**DOI:** 10.1186/s12864-022-08568-8

**Published:** 2022-05-03

**Authors:** Aijun Wang, Li Ma, Xinyue Shu, Yuqi Jiang, Juan Liang, Aiping Zheng

**Affiliations:** College of Agronomy, Sichuan Agricultural University, Chengdu, China

**Keywords:** Rice, Resistance gene, Cytochrome P450, Biosynthesis of flavonoids, Plant immunity

## Abstract

**Background:**

The sustainable development of rice production is facing severe threats by a variety of pathogens, such as necrotrophic *Rhizoctonia solani* and hemibiotrophic *Xanthomonas oryzae* pv. oryzae (*Xoo*). Mining and applying resistance genes to increase the durable resistance of rice is an effective method that can be used to control these diseases.

**Results:**

In this research, we isolated and characterized CYP716A16, which is a positive regulator of rice to *R. solani* AG1-IA and *Xoo*, and belongs to the cytochrome P450 (CYP450) protein 716A subfamily. Overexpression (OE) of *CYP716A16* resulted in enhanced resistance to *R. solani* AG1-IA and *Xoo*, while RNA interference (RNAi) of *CYP716A16* resulted in increased susceptibility compared with wild-type (WT) plants. Additionally, jasmonic acid (JA)-dependent defense responses and reactive oxygen species (ROS) were activated in the *CYP716A16*-OE lines after *R. solani* AG1-IA inoculation. The comparative transcriptomic and metabolomics analysis of CYP716A16-OE and the WT lines showed that OE of *CYP716A16* activated the biosynthesis of flavonoids and increased the amounts of narcissoside, methylophiopogonanone A, oroxin A, and amentoflavone in plants.

**Conclusion:**

Based on these results, we suggest that JA-dependent response, ROS level, multiple resistance-related proteins, and flavonoid contents play an important role in *CYP716A16*-regulated *R. solani* AG1-IA and *Xoo* resistance. Our results broaden our knowledge regarding the function of a P450 protein 716A subfamily in disease resistance and provide new insight into the molecular mechanism of rice immune response.

**Supplementary Information:**

The online version contains supplementary material available at 10.1186/s12864-022-08568-8.

## Background

Rice (*Oryza sativa* L.) is an important crop with an irreplaceable role in ensuring food security [1]. However, high and stable production of rice is severely threatened by a variety of pathogens, such as necrotrophic *Rhizoctonia solani,* which causes rice sheath blight (RSB) [2, 3], and hemibiotrophic *Xanthomonas oryzae* pv. oryzae (*Xoo*), which causes bacterial leaf blight (BLB) [4]. These two diseases are the most prevalent rice diseases worldwide [5, 6]. Mining resistance gene resources, and applying them to increase the resistance of rice is an effective method for controlling disease [7]. Recently, some genes that are resistant to these diseases have been identified, and several are provided potential solutions for applied to resistance breeding in rice [8–10]. However, the transformation of a single gene appears to have a limited effect on rice disease resistance, while in contrast, combining multiple resistance genes contributes to broad-spectrum and durable resistance to pathogens [11, 12]. Therefore, detecting new resistance genes in the rice germplasm is important for controlling disease.

In nature, plants are constantly threatened by a variety of pathogens and thus have developed complex defense systems to defend against invasion. Examples of these are the activation of the jasmonic acid (JA), salicylic acid (SA), and ethylene (ET)-related defense pathways, the up-regulation of pathogenesis-related (PR) genes; the production of reactive oxygen species (ROS), and antimicrobial peptides and phytoalexins accumulation, and callose deposition, to defend against pathogen invasion [13, 14]. For example, the NLR gene *Xa1* and receptor-like protein kinase *Xa21* imparts resistance against the *Xoo* through recognizing pathogen avirulence effectors [15, 16]. Moreover, some transcription factors, such as *OsC3H12* [17], *OsWRKY45* [18], and *OsTFX1* [19], are also involved in rice resistance to *Xoo*. Compared with well-documented studies on resistance against *Xoo*, the interaction mechanism of rice and *R. solani* AG1-IA is still very limited [20–22]. Thus, the regulation of JA-dependent defence signalling and ROS has an important role in the resistance to *R. solani* AG1-IA [23, 24]. An example is *OsRSR1* and *OsRLCK5* imparting resistance against the RSB fungus through mediated ROS levels by the glutathione-ascorbic acid antioxidant system [9].

Cytochrome P450 (CYP450) protein is one of the largest gene families in plants, and is involved in the regulation processes of a variety of secondary metabolism processes, such as those involving phytoalexin, terpenoids, and flavonoids [25, 26]. A study has indicated that CYP450 plays an important role in hormone biosynthesis and signal transduction [27]. Some of the CYP450 members are also involved in plant defense against pathogens. The cotton P450 gene *GhCYP82D* is involved in disease resistance by modulating the biosynthetic pathway of oxylipins and JA anabolism [28]. In Arabidopsis, the CYP450 protein encoding the *CYP82C2* gene plays a key role in JA-induced immunity, and overexpression (OE) of *CYP82C2* enhances the resistance to necrotrophic fungus *Botrytis cinerea* though activation of expression of JA-related defense genes [29].

Among the rice genome (http://bioinfo.cau.edu.cn/~jyyu/drcyp), 534 CYP450 proteins encoded genes were annotationed, but the vast majority of these genes have unknown functions [30]. Studies have shown that rice P450 gene *CYP71P1* possesses tryptamine 5-hydroxylase enzymatic activity and increases the resistance to *M. oryzae* through catalyzing the conversion of tryptamine to serotonin [31]. The CYP78A gene *BSR2* could positively regulates the resistance to *R. solani* in rice [32]. With the identification of genes encoding multiple types of CYP450, cloning and research to investigate the functions of each of these genes may increase our understanding of the roles of CYP450 in plant immunity.

Previously, we obtained 653 genes that exhibit significant association with the RSB resistance through genome-wide association study analysis (GWAS) analysis based on 2,888,332 high-confidence single nucleotide polymorphisms (SNPs) [9]. Among these 653 genes, the CYP450 protein encoding gene LOC_Os07g33440 was found to be strongly expressed under *R. solani* AG1-IA inoculation conditions, with high expression in the resistant cultivar Teqing as compared with the susceptible cultivar Lemont. A BLAST search against the National Center for Biotechnology Information GenBank database (NCBI) GenBank database, rice gene annotations (http://rice.plantbiology.msu.edu), and literature analysis revealed that this gene belongs to the 716A subfamily of CYP450, therefore we named it as CYP716A16. In addition, OE of CYP716A16 in rice was shown to confer enhanced tolerance to *R. solani* AG-IA and *Xoo* pathogens compared to the control plants, while RNA interference (RNAi) of *CYP716A16* promoted the colonization of these two pathogens in rice. Furthermore, OE of *CYP716A16* was induced increased expression of PR and JA-related defense genes. Comparative transcription analysis of wild type (WT) and CYP716A16-OE plants suggested that *CYP716A16* regulated the accumulation of flavonoids and activation of the phytoalexin synthesis-related genes. From these results, we conclude that *CYP716A16* plays a key role in regulating rice immunity.

## Results

### CYP716A16 was significantly associated with *R. solani* AG1-IA and *Xoo* resistance

**Fig. 1:**
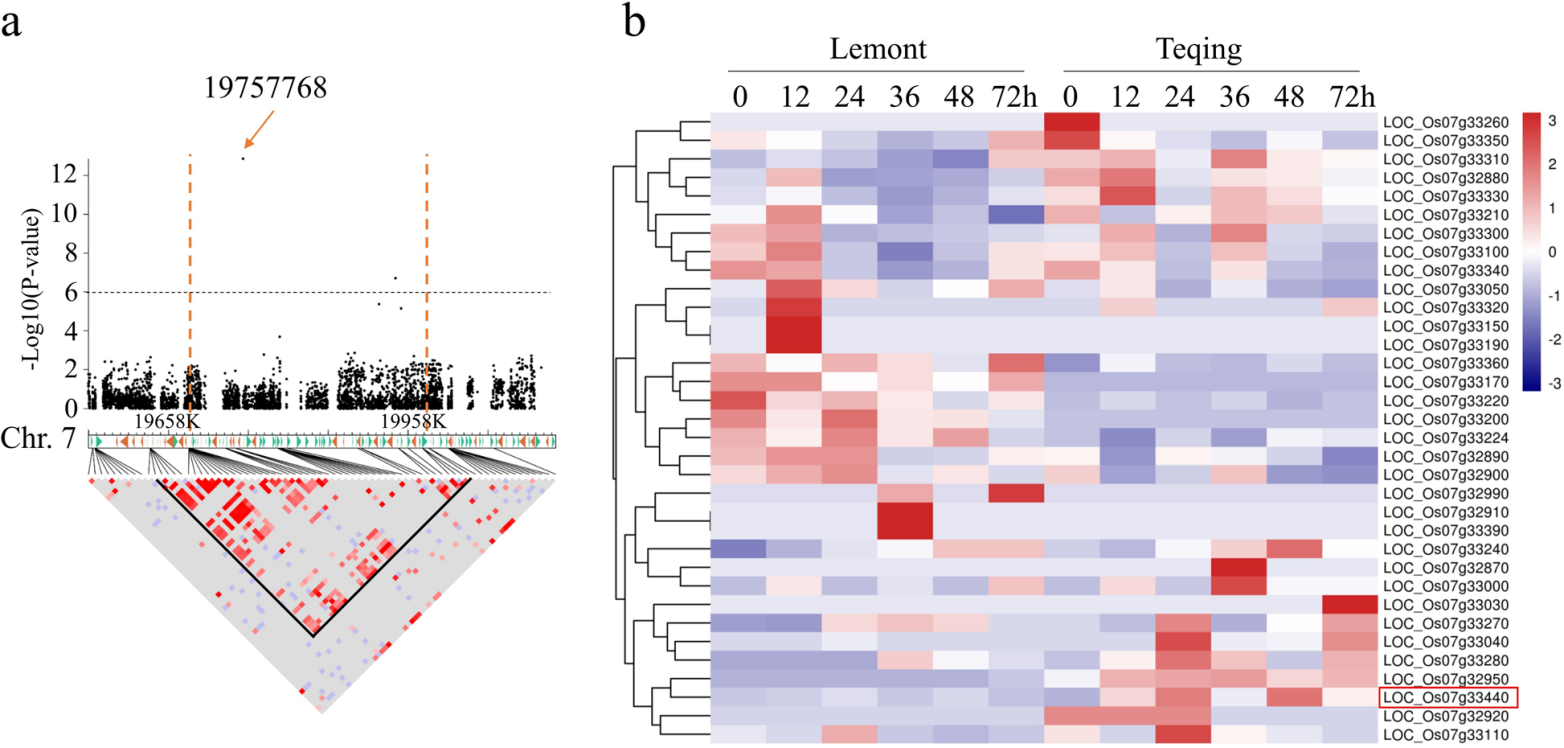
The *CYP716A16* was significantly associated with rice sheath blight (RSB) resistance, and its transcription was induced by the infection of *Rhizoctonia solani* AG1-IA. **a** Local Manhattan plot of single nucleotide polymorphism (SNP)-based association (top) and LD heatmap (bottom) surrounding the peak on chromosome 7. Red dashed lines indicate the candidate region for associated SNPs; **b** Expression of 58 candidate genes in resistant and susceptible rice lines at different inoculation times (transcriptome data)

In our previously reported, 653 genes that associate with RSB resistance were obtained through SNP-based and haplotype-based GWAS combined with transcriptome analysis of resistant and susceptible varieties [9]. Among these 653 genes, CYP450 protein encoding gene *LOC_Os07g33440* (named *CYP716A16*) was associated with RSB resistance and located on a 290 kb (19,688-19,978 Kb) haplotype block, based on a significant association with SNP loci Chr. 7_19757768 (−log10P = 12.86) (Fig. 1a). This haplotype block encompassing 58 genes (Fig. 1a), and the expression pattern of these 58 genes in the resistant variety Teqing and susceptible variety Lemont, are shown in Fig. 1b. The expression of *CYP716A16* dramatically increased after *R. solani* AG1-IA infection at different hours post-inoculation (hpi) in Teqing, whereas the expression level of this gene in the Lemont variety was nearly unchanged with inoculation time, as compared with 0 hpi (Fig. 1b). Interestingly, *CYP716A16* was also associated with BLB resistance based on GWAS analysis, and up-regulation was induced by *Xoo* infection in the resistant variety NSIC RC154, although it was not induced in susceptible variety CT 9737-6-1-3P-M [10]. These results suggested that CYP716A16 may be involved in the process of RSB and BLB resistance.

### CYP716A16 protein belongs to the cytochrome P450 716A subfamily protein

**Fig. 2:**
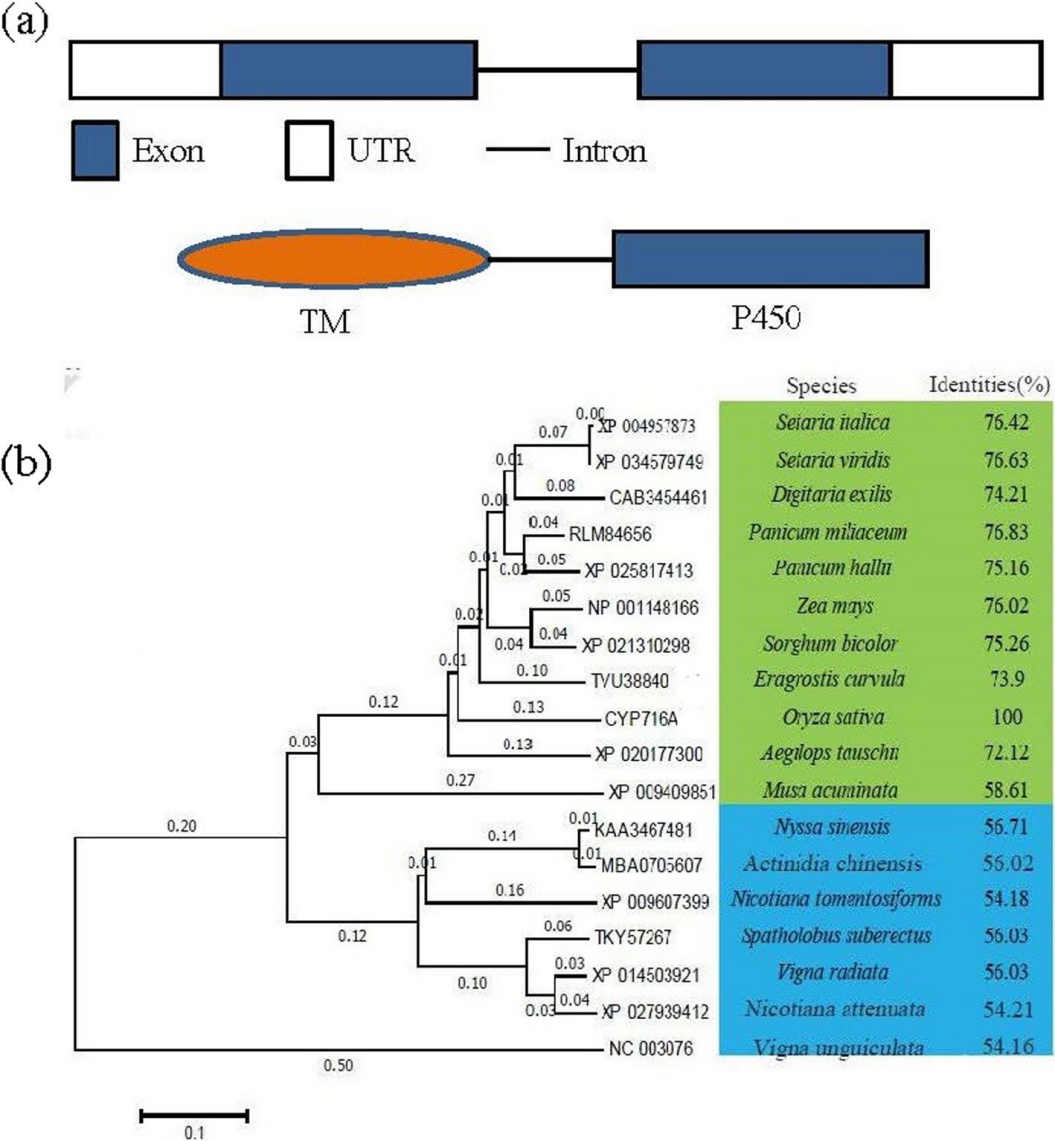
CYP716A16 encodes a cytochrome P450 protein and is ubiquitous in land plants. **a** Diagram of conserved domains in the CYP716A16 protein sequence; **b** Phylogeny of *CYP716A16* homologues in parts of plant species. The evolutionary analysis was constructed in MEGA 6.0 using the neighbor-joining method

The complete coding sequence (CDS) of *CYP716A16* (1479 bp) was obtained from a cDNA library of *R. solani* AG1-IA-inoculated rice (Teqing variety) by amplifying the cDNA ends. Sequence analysis showed that the CDS of *CYP716A16* contains two exons and one intron in the rice genome, and conserved structural analysis indicated that *CYP716A16* contains a P450 domain, with the transmembrane domain (TM) was also being predicted (Fig. 2a). The putative homologues were identified by BLASTP searches with rice *CYP716A16* as the query, and the proteins were aligned through ClustalW2. A phylogenetic tree was constructed using MEGA 6.0 (Fig. 2b), revealing that the protein shares high homology with the CYP450 716A subfamily proteins of monocotyledonous plants such as *Eragrostis curvula*, *Sorghum bicolor*, *Zea mays*, *Panicum hallii*, *Panicum miliaceum*, *Digitaria exilis*, *Setaria viridis*, and *Setaria italic*, and it is distantly related to the CYP450 716A subfamily proteins of dicotyledonous plants such as *Gossypium australe*, *Nicotiana tomentosiformis*, *Musa acuminate*, and *Spatholobus suberectus*. Based on these results, the CYP716A16 was identified as a CYP450 716A subfamily protein, and it is conserved in land plants.

### Overexpression of *CYP716A16* enhanced rice resistance to *R. solani* AG1-IA and *Xoo*

**Fig. 3:**
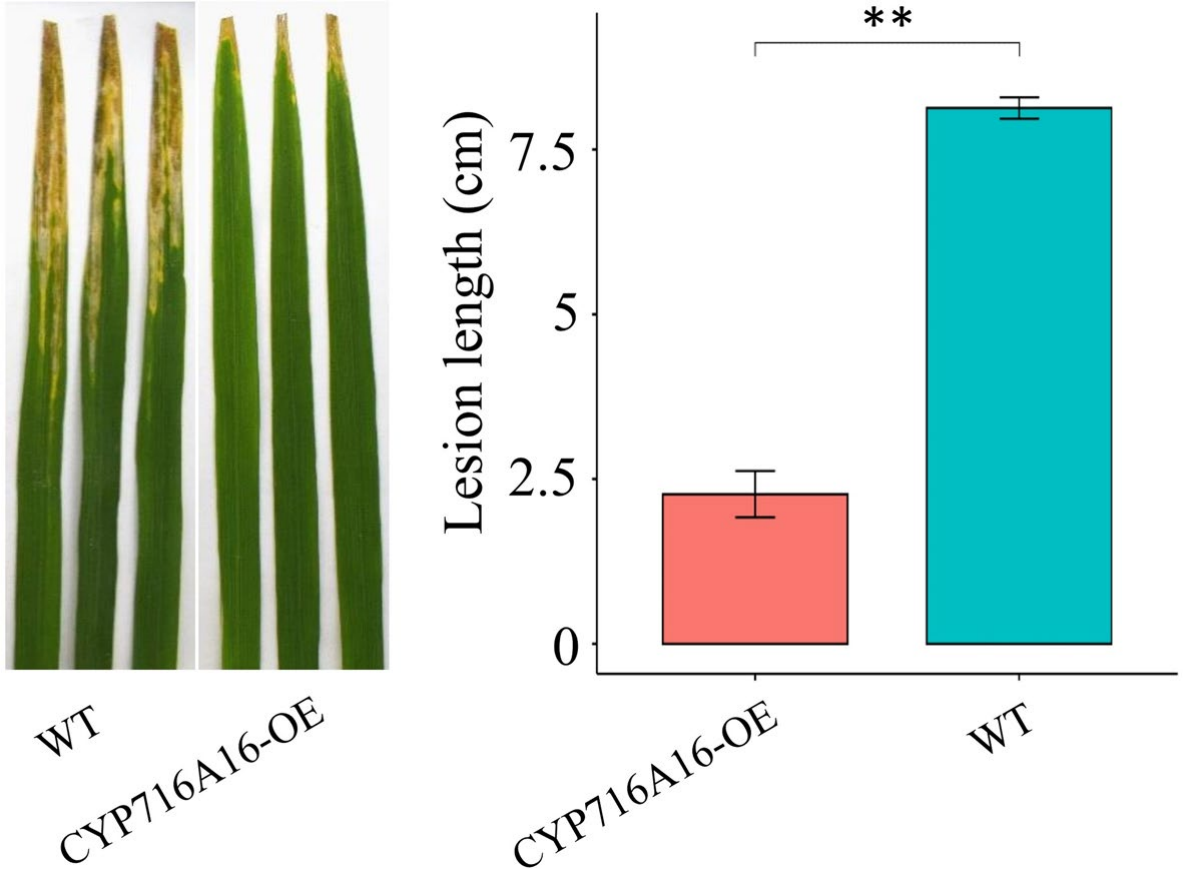
Overexpression (OE) of *CYP716A16* in rice suppressed infection by *R. solani* AG1-IA and *Xoo*. The leaves of CYP716A16-OE plants were challenged with *Xoo* and were more resistant compared to the WT. Three independent experiments were performed (** *P* < 0.01)

To investigate the function of *CYP716A16* in rice immunity, the OE transformation construct 35S::CYP716A16 was generated and transformed into the Nipponbare background. qRT-PCR results confirmed the high transcription level of *CYP716A16* in four homozygous OE lines, CYP716A16-OE2, CYP716A16-OE4, CYP716A16-OE5, and CYP716A16-OE9 [9]. We used stable T_1_ progenies of four OE lines for disease resistance evaluation. Detached rice leaves from the CYP716A16-OE plants at tillering stage challenged with *R. solani* AG1-IA showed that growth of *R. solani* AG1-IA was greatly suppressed in CYP716A16-OE lines relative to WT Nipponbare [9]. Furthermore, to investigate whether CYP716A16 affected rice resistance to BLB, we performed inoculation experiments with the highly pathogenicity *Xoo* race P6. The results showed that the CYP716A16-OE lines exhibited an enhanced resistance to the *Xoo* race P6. There were significantly shorter lesions on CYP716A16-OE as compared to the WT (Fig. 3). Importantly, there were no significant differences in agronomic traits between CYP716A16-OE and WT lines (Supplementary Fig. S1). These results suggested that *CYP716A16* positively regulates the rice resistance response to RSB and BLB, and plays a positive role in rice basal disease resistance against the fungal and bacterial pathogen.

### RNA interference of CYP716A16 decreases rice resistance to *R. solani* AG1-IA and *Xoo*

**Fig. 4:**
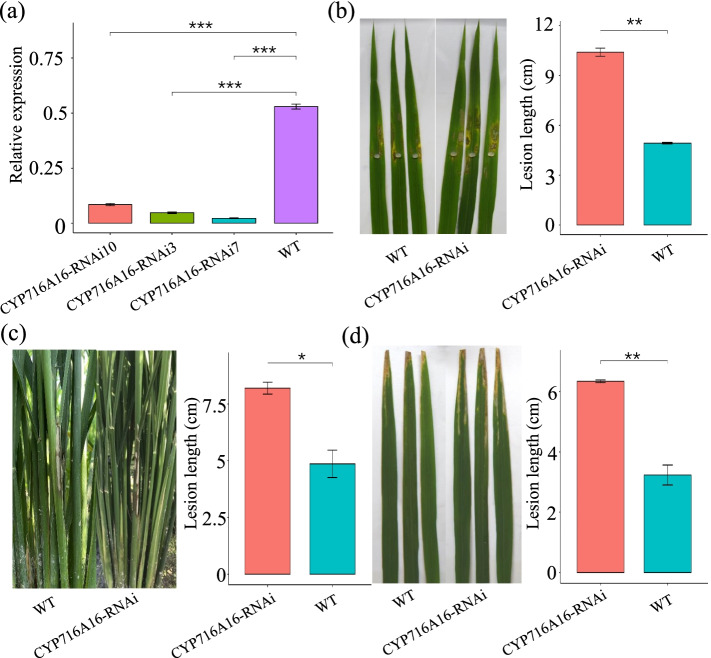
Interference of *CYP716A16* genes enhanced rice susceptibility to *R. solani* AG1-IA and *Xoo*. **a** The qRT-PCR results indicate that *CYP716A16* was significantly downregulated in the RNA interference (RNAi) lines. Statistical analysis was determined by one-way ANOVA followed by Tukey’s multiple comparison tests (***P < 0.001); **b** Detached leaves of *CYP716A16-*RNAi plants were challenged by *R. solani* AG1-IA, and the lesion lengths were measured at 3 days post-inoculation (dpi). Three independent experiments were performed (** *P* < 0.01); **c** RNAi of *CYP716A16* genes enhanced the rice susceptibility to *R. solani* AG1-IA in the field (*P < 0.05); **d** The leaves of CYP716A16-RNAi plants were challenged with *Xoo* and were more susceptible compared to the WT. Three independent experiments were performed (** *P* < 0.01)

To further determine the function of *CYP716A16* in defense against pathogens, we obtained transgenic lines with decreased expression of *CYP716A16* in a Teqing (with moderately resistance to *R. solani*) background. The reduced expression was achieved by RNAi, and the lower transcription level of *CYP716A16* in three homozygous RNAi lines, CYP716A16i3, CYP716A16i7, and CYP716A16i10, were verified by qRT-PCR (Fig. 4a). Then, these CYP716A16-RNAi plants were inoculated with *R. solani* AG1-IA to evaluate the defensive role of CYP716A16. The lesion lengths were measured at 3 d after inoculation, and CYP716A16-RNAi exhibited significantly longer lesions than the WT Teqing (Fig. 4b). In a field measure of resistance against *R. solani* AG1-IA, we found that the disease degree of the CYP716A16-RNAi transgenic line was significantly higher than that of WT (Fig. 4c). Similarly, we performed inoculation experiments with *Xoo* race P6. We found that the CYP716A16-RNAi lines exhibited an enhanced susceptibility to the *Xoo* strain P6 (Fig. 4d). We also found that the agronomic traits were not significantly different between CYP716A16-RNAi and WT plants (Supplementary Fig. S1). These results indicated that the RNAi of *CYP716A16* in Teqing plants impaired resistance to *R. solani* AG1-IA and *Xoo*.

### Overexpression of CYP716A16 in rice leads to induced expression of defense-related genes

**Fig. 5:**
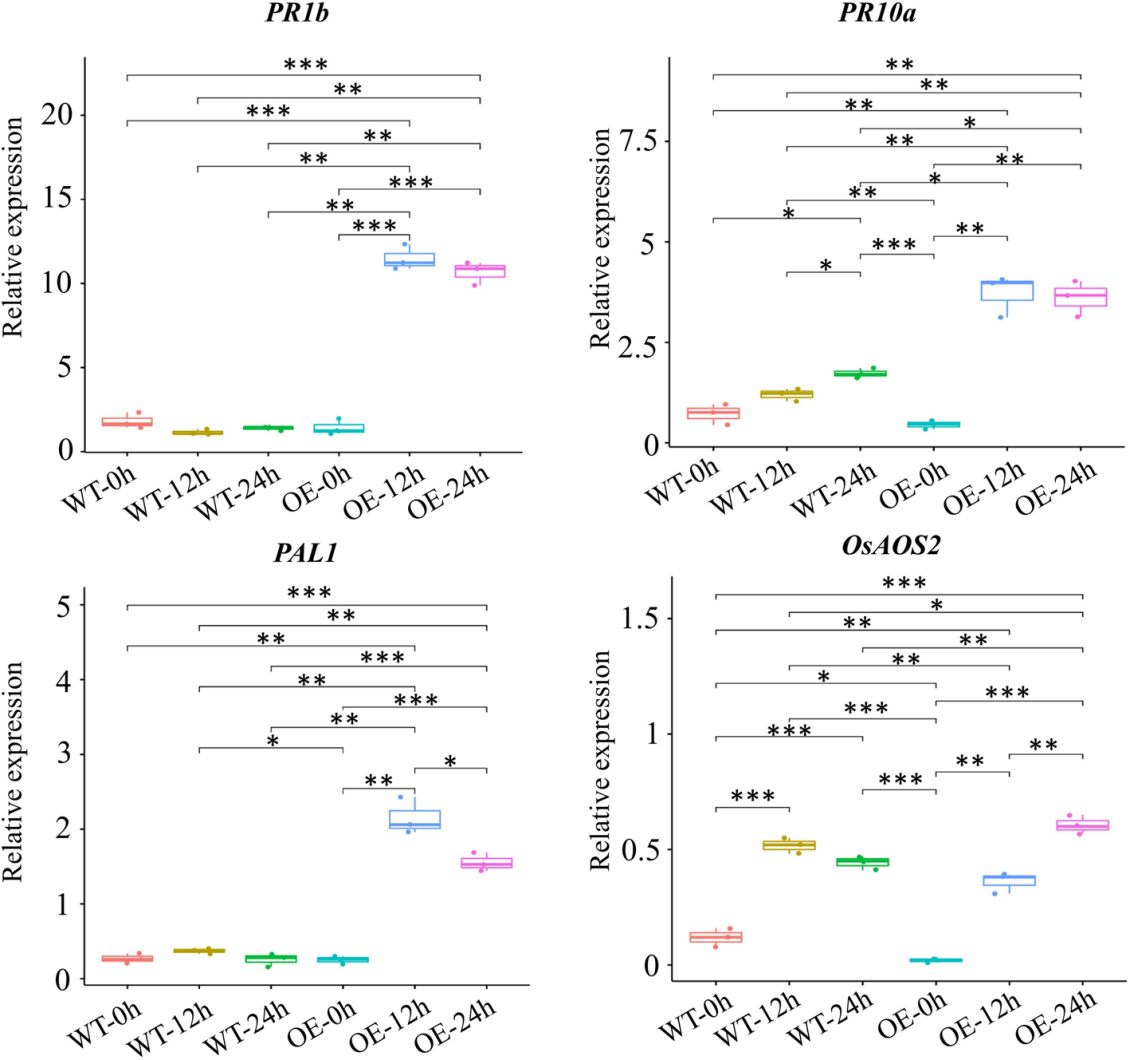
OE of *CYP716A16* activating the jasmonate acid (JA) biosynthetic and pathogenesis-related (PR) genes. The relative expression levels of the tested genes were normalized to UBQ. Three independent experiments were performed for each test of the gene expression levels. Statistical analysis was determined by one-way ANOVA, followed by Tukey’s multiple comparison tests (*P < 0.05; ** *P* < 0.01; *** *P* < 0.001)

We examined the PR genes transcription levels in CYP716A16-OE plants and WT during a time course of 0–24 h (0, 12, 24 h) in leaves inoculated with *R. solani* AG1-IA. In CYP716A16-OE plants, these three genes were constitutively expressed and were further induced to significantly higher levels than those in the WT plants (Fig. 5). We also identified the expression of JA (*OsAOC*, *OsAOS2*), ET (*OsACS2*), and SA (*OsNPR1*) biosynthesis or signaling-related genes in CYP716A16-OE plants and WT at 0, 12, and 24 hpi [33–36]. The results showed that pathogen challenge strongly induced the expression of OsAOS2 in the WT and CYP716A16-OE, and the expression levels in the CYP716A16-OE lines were significantly higher than those in the WT at 24 hpi. Interestingly, the expression of OsAOC was only strongly induced in CYP716A16-OE plants at 24 hpi (Supplementary Fig. S2). In contrast, the expression of the ET signaling-related genes *OsACS2* was decreased (Supplementary Fig. S2). Additionally, *OsNPR1*, a marker gene of SA-induced resistance response, showed no significantly difference between CYP716A16-OE plants and the WT (Supplementary Fig. S2).

Furthermore, we did not observe significant differences between ET and JA levels among the WT and CYP716A16-OE plants. Only the level of SA was significantly increased in CYP716A16-OE plants compared with WT (Supplementary Fig. S3). Additionally, JA-isoleucine (JA-Ile) is the active form in JA signal transduction, and thus, we detected JA-Ile in CYP716A16-OE and WT plants, and the amount of JA-Ile in CYP716A16-OE plants was higher than that in the WT (Supplementary Fig. S3). Therefore, we concluded that the defense responses-related to JA were involved in disease resistance regulated by CYP716A16.

### CYP716A16 modulates ROS and related antioxidant enzymatic activity

**Fig. 6:**
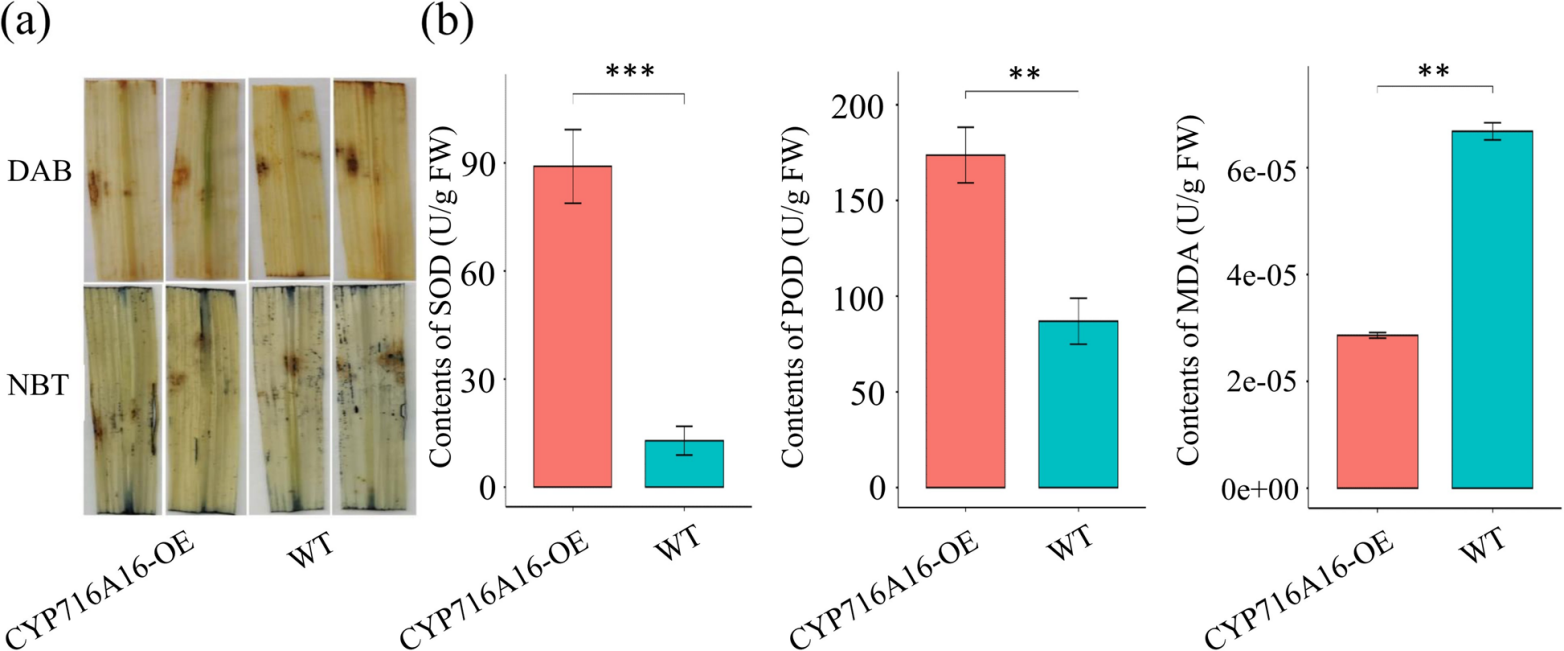
OE of *CYP716A16* activating the ROS burst and related antioxidant enzymatic activity. **a** The levels of H_2_O_2_ and O_2_^−^ in CYP716A16-OE and WT plants were assayed using DAB and NBT staining at 24 hours post-inoculation (hpi); **b** The amounts of superoxide dismutase (SOD), peroxidase (POD), and malondialdehyde (MDA) in CYP716A16-OE and WT plants were measured at 24 hpi. Three biological repetitions were performed (** *P* < 0.01; *** *P* < 0.001)

H_2_O_2_ and O_2_^−^ are primary ROS that function as positive signaling molecules and played an important role in disease resistance in plants [37]. To explore whether ROS burst is involved in the defense response mediated by *CYP716A16* in rice, levels of H_2_O_2_ and O_2_^−^ were assayed using DAB and NBT staining, respectively. At 24 h after *R. solani* AG1-IA infection, additional DAB- and NBT- stained spots appeared on the leaves surrounding the lesions on CYP716A16-OE plants as compared to WT plants (Fig. 6a), suggesting that CYP716A16-OE increased the accumulation of H_2_O_2_ and O_2_^−^ in the transgenic rice. Furthermore, superoxide dismutase (SOD), peroxidase (POD), and malondialdehyde (MDA) are three important antioxidant enzymes in the ROS-scavenging system, which is critical for maintaining ROS homeostasis in plants [38]. Thus, we detected the activity levels of SOD, POD, and MDA content in CYP716A16-OE and WT plants. From the results, it was observed that the activation of POD and SOD in CYP716A16-OE plants was significantly elevated compared to the WT at 24 h after *R. solani* AG1-IA infection, whereas the activation of MDA was reduced (Fig. 6b). These data suggested that *CYP716A16* was involved in rice immunity response through mediated ROS burst.

### Biosynthesis of flavonoids was activated in the CYP716A16-OE plant

**Fig. 7:**
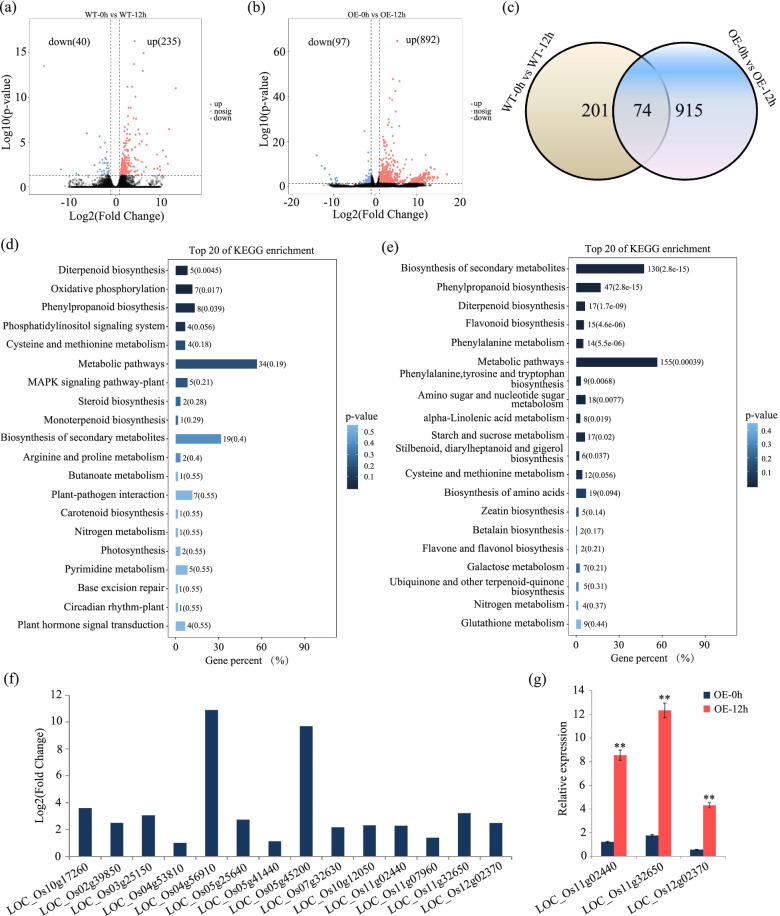
Analysis of the RNA-Seq data for CYP716A16-OE and WT plants. **a** The number of differentially expressed genes (DEGs) in WT plants at 12 hpi; **b** The number of DEGs in CYP716A16-OE plants at 12 hpi; **c** Venn diagrams showing the overlap of DEGs in CYP716A16-OE and WT plants at 12 hpi; **d** The KEGG enrichment analysis of the DEGs in WT plants. The sizes and the colours of the dots represent the numbers of DEGs and the *p*- value, respectively; **e** The KEGG enrichment analysis of the DEGs in CYP716A16-OE plants. The sizes and the colours of the bars represent the numbers of DEGs and the q- value, respectively; **f** The expression of 15 DEGs related to the biosynthesis of flavonoids in CYP716A16-OE plants. Three of them are involved in the phytoalexin biosynthesis; **g** Expression analysis of three phytoalexin biosynthesis-related genes in CYP716A16-OE plants at 12 hpi by qRT–PCR

To explore the roles of *CYP716A16* in the defense against pathogens, we performed RNA-Seq using uninfected leaves and leaves of the WT and CYP716A16-OE lines that had been infected with *R. solani* AG1-IA for 12 h. The sequence number information for the RNA-Seq data is shown in Supplementary Table 1. The transcriptome data indicated that there are 40 genes down-regulated and 235 genes up-regulated in WT-12hpi compared to WT-0hpi (Fig. 7a; Supplementary Table 2). However, the OE of CYP716A16 resulted in increased expression of 892 genes and decreased expression of 97 genes (Fig. 7b; Supplementary Table 2). Additionally, 74 differentially expressed genes (DEGs) were shared by the two lines at 12 hpi, and 915 DEGs were uniquely detected in CYP716A16-OE plants (Fig. 7c). Kyoto Encyclopedia of Genes and Genomes (KEGG) assay revealed that these DEGs were assigned into several functional classes, and among which biosynthesis of flavonoids was significantly enriched (*P* < 0.05) in CYP716A16-OE lines (Fig. 7e), but no significant enrichment was found in the WT (Fig. 7d). In this class, there are 15 genes putatively related to biosynthesis of flavonoids were uniquely up-regulated in CYP716A16-OE lines (Fig. 7f), including *LOC_Os01g53370* (anthocyanidin 5,3-O-glucosyltransferase), *LOC_Os02g39850* (transferase family protein), *LOC_Os05g45200* (anthocyanidin 5,3-O-glucosyltransferase), *LOC_Os03g25150* (transposon protein), *LOC_Os04g53810* (leucoanthocyanidin reductase), *LOC_Os04g56910* (transferase family protein), *LOC_Os05g25640* (cytochrome P450), *LOC_Os05g41440* (cytochrome P450), *LOC_Os11g07960* (transferase family protein), *LOC_Os07g32630* (UDP-glucoronosyl and UDP-glucosyl transferase domain containing protein), *LOC_Os10g12050* (expressed protein), *LOC_Os10g17260* (cytochrome P450), *LOC_Os11g02440* (chalcone--flavonone isomerase), *LOC_Os11g32650* (chalcone synthase), and *LOC_Os12g02370* (chalcone--flavonone isomerase). Among these 15 genes, the transcript levels of three genes related to phytoalexin biosynthesis (*LOC_Os11g02440*, *LOC_Os11g32650*, and *LOC_Os12g02370*) in CYP716A16-OE lines were validated by qRT-PCR (Fig. 7g). The results indicated that *CYP716A16* may activate flavonoid biosynthesis, with the subsequent result of disease resistance.

Furthermore, we determined the different of metabolites in the WT and CYP716A16-OE lines at 12 hpi. In total, 1218 metabolites were detected in rice by liquid chromatography mass spectrometery (LC-MS), including many primary and secondary metabolites, such as amino acids, fatty acyls, phytohormones, sugar alcohols, flavonoids, quinones, and terpenoids (Supplementary Table 3). Compared with the control (0 h), 214 measured metabolites were identified as significantly increased (fold change≥2) in WT plants at 12 h after *R. solani* AG1-IA inoculation. However, only 51 significantly increased metabolites were observed in CYP716A16-OE plants (Supplementary Table 3). Interestingly, some measured metabolites showed exclusively significant increases in CYP716A16-OE plants that underwent *R. solani* AG1-IA inoculation, including four flavonoids (narcissoside, methylophiopogonanone A, oroxin A, and amentoflavone) and trans-zeatin (Supplementary Table 3). From these results, we concluded that these metabolites may play important roles in disease resistance mediated by *CYP716A16*.

### Overexpression of CYP716A16 promoted accumulation of phytoalexin

From the transcriptome data, we found several key genes, which involved in phytoalexin biosynthesis that were up-regulated in CYP716A16-OE plants at 24 h after *R. solani* AG1-IA inoculation, although with no significantly different expression in the WT. This indicated that phytoalexin was involved in the process of disease resistance regulated by *CYP716A16*. To further confirm this result, we measured the amounts of momilactone A and B, which are important phytoalexins involved in rice defense, in WT and CYP716A16-OE plants. There was a significant increase in the amounts of momilactone B in CYP716A16-OE plants as compared with the WT at 24 hpi (Supplementary Fig. S4).

## Discussion

The characterization of resistance genes in rice is basic to the development of rice varieties with disease resistance. Bioinformatics methods, such as GWAS, bulk segregant analysis (BSA), and transcriptomic analyses, have been used to detect the genes that control crop resistance to pathogen infection [1, 39–41]. In our previous study, a GWAS was performed using 259 diverse rice germplasm with genotypes based on SNPs and haplotypes with their RSB reactions at three developmental stages, seedlings, tillering, and booting was performed. Furthermore, we performed a combined comparative transcriptomic analysis between Teqing (a line that is resistant line to *R. solani* AG1-IA) and Lemont (a line that is susceptible to *R. solani* AG1-IA) after *R. solani* AG1-IA infection, and we obtained 653 core candidate genes that might regulate rice resistance to RSB [9]. In this study, we selected the CYP450 protein encoding gene CYP716A16 to verify its resistance function, and this further refined our results.

In plants, the CYP450 proteins are divided into 10 separate clans in 61 families [42]. In this study, we isolated and characterized a novel CYP450 gene, *CYP716A16*, which belongs to the CYP716A subfamily, in rice. *CYP716A16* is a widespread family with diversity in structure and function in plants, and members of the CYP716A family are involved in triterpene biosynthesis [43, 44]. For example, licorice CYP450 monooxygenase *CYP716A179* plays a key role in the biosynthesis of oleanolic acid and betulinic acid [43]. Although the role of more than a dozen CYP716As in triterpene biosynthesis has been identified, little information is available on the disease resistance functions of the CYP716A subfamily in plants [43]. Here, we show that OE of the *CYP716A16* enhanced the resistance of rice to necrotrophic *R. solani* AG1-IA and hemibiotrophic *Xoo*. Conversely, the resistance level in CYP716A16-RNAi rice plants was significantly reduced. These results provide new insight into the function of the P450 716A subfamily.

Plant hormones, especially JA, ET, and SA, play important roles in the regulation of plant innate immunity [14]. Additionally, JA-dependent plant immunity plays a key role in resistance to necrotrophic and hemi-necrotrophic fungus [9]. For instance, JA and ET are both involved in the resistance of *R. solani* AG1-IA in rice, and are mediated by OsWRKY4 and OsWRKY80 [45]. Kouzai et al. [46] found that foliar pretreatment with SA can induce sheath blight resistance in rice and *Brachypodium distachyon*. In our study, genes related to JA biosynthesis, such as OsAOC and OsAOS2, were upregulated in the CYP716A16-OE plants. Furthermore, the JA response gene PR1b was up-regulated in the CYP716A16-OE plants, indicating that the JA signalling pathway is activated in CYP716A16-OE plants. Therefore, we suggest that activation of a JA-dependent defense in CYP716A16-OE contributes to rice resistance against *R. solani* AG1-IA and *Xoo*.

Flavonoids are secondary metabolites that occur widely occur in plants, and can be divided into subgroups including anthocyanidins, flavonols, flavones, flavanols, flavanones, chalcones, dihydrochalcones and dihydroflavonols [47, 48]. Previous studies have shown that flavonoid biosynthesis is an important pathway during the interaction between *Medicago truncatula* and *R. solani* [49]. In rice, kaempferol, naringenin, and dihydroquercetin are involved in the defense against the fungal blast pathogen *Pyricularis oryzae* [50, 51]. We show in this study that the biosynthesis of flavonoids was significantly enriched in CYP716A16-OE plants at 12 hpi, and furthermore, there were increases in the amounts of narcissoside, methylophiopogonanone A, oroxin A, and amentoflavone, which is preliminary proof that the flavonoids are regulated by a CYP716A16. Moreover, some genes involved in phytoalexin were also induced up-regulated in CYP716A16-OE plants. Thus, from these results and the results of transgenic experiments in this study, we showed that CYP716A16 participates in flavonoids biosynthesis and that it plays a positive regulatory role in the resistance to *R. solani* AG1-IA and *Xoo* in rice.

Even though there are several RSB resistance genes have been cloned, none of them have been used in rice breeding for RSB resistance. CYP716A16 encodes a CYP450 proteins, and OsBON1 and OsBON3 are copine genes [52]. Similar to OsBON1 and OsBON3, CYP716A16 also confers broad-spectrum disease resistance, constituting a superior disease-resistant characteristic for rice breeding. The CYP716A16 gene, however, carries a further advantage over OsBON1. The RNAi of OsBON1 increases the disease resistance of rice with a decrease in the tillers number [52], and thus, the yields are affected. In contrast, our measurements suggest that CYP716A16 positively regulates resistance to disease without compromising fitness. Therefore, CYP716A16 represents a more optimal alternative genetic resource for rice resistance breeding in rice.

## Conclusions

Our findings indicate that the CYP450 protein encoding gene *CYP716A16* positively contributes to the immune response in rice, which will expand our understanding of the potential functions of P450 proteins and provide valuable insight into the molecular mechanism of plant immunity. Furthermore, the *CYP716A16* displays broad-spectrum disease resistance to both bacterial and fungal pathogens, and this will also provide important gene resources for rice disease resistance breeding in rice.

## Materials and methods

### Plant materials and growth conditions

The rice cultivars Teqing, Lemont, and Nipponbare were used in this work. All plant materials were planted in the rice transgenic field of the College of Agronomy, Sichuan Agricultural University, Chengdu.

### Vector construction and rice transformation

To construct the Nipponbare CYP716A16*-*OE plants, the CDS of *CYP716A16* was amplified from the cultivar Teqing by PCR using the gene-specific primers (Supplementary Table 4). The cDNA product was then inserted into *pBWA(V)HS*, which harbors a cauliflower mosaic virus (CaMV) 35S promoter, and the constructed vector was introduced into *Agrobacterium tumefaciens* GV3101. Rice transformation was performed following the methods previously described [53]. Transgenic plants of the T_1_ generation with positive activity were used in the experiments. For RNAi vector construction, a specific *CYP716A16* fragment of approximately 270 bp was selected, and then amplified with the primers listed in Supplementary Table S4. The plasmid was constructed as previously described [54, 55]. The CYP716A16-RNAi cis- and trans-fragments with correct sequences were inserted into the vector *pBWA(V)HS*. *Agrobacterium tumefaciens*-mediated transformation of Teqing was used to obtain the CYP716A16-RNAi transgenic plants. Transgenic plants of the T_1_ generation with positive activity were used in the experiments.

### Pathogenic infection

For *R. solani* AG1-IA, we identified the resistance level of rice plants in indoor and field inoculation. The second youngest leaf from the main tiller was cut around the heading stage and inoculated into a 5 mm potato dextrose agar (PDA) plug containing *R. solani* AG1-IA mycelia, placed on a moistened filter paper, and maintained in a petri plate. To enhance humidity and increase *R. solani* AG1-IA infection and development, the moisture of the filter paper was maintained with sterile water, and the plate was covered with protective film. After 72 h, the length of each leaf lesion was measured. In the field, the pathogen was grown on truncated thin matchsticks (0.8–1.0 cm long 2–3 mm wide, and 1 mm thick) on potato dextrose broth medium at 28 °C in the dark for 2-3 d. To perform inoculation, the inoculum was closely affixed to one side of the base of the seedling stem, assuring that hypha was directly touching the plant [39]. Five sheaths per line were inoculated as replications.

For *Xoo*, we used *Xoo* virulent strains P6 to artificially inoculate plants. At the rice tillering stage, 15 of the uppermost leaves of each variety were inoculated with the *Xoo* race P6, using the leaf-clipping method [56]. Lesion lengths were measured on all inoculated leaves at 14 days post-inoculation (dpi), when lesions were easily visible as well as stable.

### qRT-PCR analysis

Plant total RNA was extracted using a Plant Total RNA Isolation Kit (Sangon Biotech, Shanghai, China). First strand cDNA was synthesized from total RNA using the Transcriptor First-Strand cDNA Synthesis Kit (Roche, Indianapolis, IN, USA). The cDNA samples were then subjected to qRT-PCR on a Bio-Rad CFX96 Real-Time PCR System (Foster City, CA, USA), according to the manufacturer’s instructions. The PCR reactions were prepared in a 20 μL volume, containing 3 μL cDNA and 1 μL each of the forward and reverse gene specific primers. Each PCR was replicated four times. The ubiquitin (UBQ) gene was used as an internal control for data normalization. Gene expression levels were calculated using the 2^-∆∆Ct^ method. The primers used for qRT-PCR are listed in Supplementary Table 4.

### H_2_O_2_ measurement and antioxidant enzymatic activity detection

To compare the differences in ROS among WT and OE lines, the quantities of H_2_O_2_ and O_2_^−^ were measured using DAB and NBT staining, respectively, at 72 hpi according to a previously described protocol [57]. The same samples used for H_2_O_2_ quantification were used for SOD, POD, and MDA activity analysis. Total SOD, POD, and MDA activity was measured using previously described methods [58, 59].

### RNA-Seq and data analyses

Leaves of WT and CYP716A16-OE lines were harvested at 12 hpi. Leaves of each line uninfected at 12 h served as a control. RNA samples were sent to Beijing Novogene Biological Technology Co., Ltd. for cDNA library construction and Illumina sequencing (HiSeq TM 2500, San Diego, NEB, USA). RNA-Seq libraries were constructed using NEBNext® Ultra™ RNA Library Prep Kit for Illumina® (NEB, USA), according to the manufacturer’s instructions, and unique index codes were added to each sample. The sequencing was performed using an Illumina Hiseq platform to generate 125 bp paired-end sequences. Sequences with a low quality score and those containing adaptor sequences and stretches of -Ns were removed from the raw data. The reference genome of Nipponbare rice and gene model annotation files (Rice Annotation Project) were directly downloaded from the rice genome website (ftp://ftp.ensemblgenomes. org/ pub/ plants/ release_36/ fasta/ oryza_indica/ dna/). An index of the reference genome was built using Bowtie v2.2.3, and paired-end sequences were aligned to the reference genome using TopHat v2.0.12 [60–62]. The number of sequences mapped to each gene was counted using HTSeq v0.6.1 [63], and the number of fragments per kilobase of transcript sequence per million (FPKM) of each gene was calculated based on the length of the gene and number of sequence counts mapped to that gene. The differential expression gene (DEG) analysis (q < 0.05 and |log2 (fold change)| > 1) was conducted through the DEGSeq R package [64]. The Benjamini and Hochberg method was used to adjust *P*-values [65].

### KEGG enrichment analysis of DEGs

The KOBAS (v2.0) software was used for statistical enrichment analysis of DEGs in KEGG pathways [66, 67]. The hypergeometric test was performed using the ‘phyper’ function in R. KEGG terms with *P* < 0.05 were defined as significantly enriched in DEGs.

### Metabolome analyses

The same samples used for RNA-seq were used for metabolome analysis. Samples were analyzed using QTRAP 6500plus LC-MS platforms (AB SCIEX, Boston, MA, USA). Analytical conditions were based on the procedures as described in Wang et al. [68]. Quantification of metabolites was carried out using a multiple reaction monitoring method [69]. Metabolites with significant differences in content were set with thresholds of fold change ≥2 or ≤ 0.5 [70].

### Statements

The rice lines used in this study were provided by Sichuan Agricultural University and comply with relevant institutional, national, and international guidelines and legislation. Our study was approved by Sichuan Agricultural University.

## Supplementary Information

**Additional file 1: Supplementary Figure S1**. The data of important agronomic traits of WT, CYP716A16-OE, CYP716A16-RNAi lines. **Supplementary Figure S2**. The expression pattern of OsAOC, OsACS2, and OsNPR1 in WT and CYP716A16-OE plants after inoculation with *R. solani* AG1-IA. **Supplementary Figure S3**. The levels of (JA, JA-Ile, SA, and ET) in WT and CYP716A16-OE plants after inoculation 24 h with *R. solani* AG1-IA. **Supplementary Figure S4**. The contents of phytoalexin (MA and MB) in WT and CYP716A16-OE plants after inoculation 24 h with *R. solani* AG1-IA.

**Additional file 2: Supplementary Table S1**. The sequence data for the transcriptome.

**Additional file 3: Supplementary Table S2**. Differentially expressed genes between WT and CYP716A16-OE lines.

**Additional file 4: Supplementary Table S3**. Differentially expressed metabolites in the leaves of the WT and CYP716A16-OE lines.

**Additional file 5: Supplementary Table S4**. The primers sequences used in this study.

## Data Availability

All the data generated or analysed during this study were included in this article and its additional data files. The *O. sativa* transcriptome datasets analyzed during the current study are available in the National Center for Biotechnology Information (https://www.ncbi.nlm.nih.gov) under the accession number: PRJNA777627. The rice samples used in this study were deposited at College of Agronomy, Sichuan Agricultural University, Chengdu, China.
