# Supplementary material for: Rice (Oryza sativa L.) cytochrome P450 protein 716A subfamily CYP716A16 regulates disease resistance
Source: BMC Genomics. 2022 May 3;23:343. doi: 10.1186/s12864-022-08568-8 (PMC9066777; doi:10.1186/s12864-022-08568-8)
Supplement: Supplementary file 1 — Additional file 1: Supplementary Figure S1. The data of important agronomic traits of WT, CYP716A16-OE, CYP716A16-RNAi lines. Supplementary Figure S2. The expression pattern of OsAOC, OsACS2, and OsNPR1 in WT and CYP716A16-OE plants after inoculation with R. solani AG1-IA. Supplementary Figure S3. The levels of (JA, JA-Ile, SA, and ET) in WT and CYP716A16-OE plants after inoculation 24 h with R. solani AG1-IA. Supplementary Figure S4. The contents of phytoalexin (MA and MB) in WT and CYP716A16-OE plants after inoculation 24 h with R. solani AG1-IA. [file 12864_2022_8568_MOESM1_ESM.docx]

**Rice (*Oryza sativa* L.) cytochrome P450 protein 716A subfamily CYP716A16 regulates disease resistance**

Aijun Wang^1#^, Li Ma^1#^, Xinyue Shu^1^, Yuqi Jiang^1^, Juan Liang^1^, Aiping Zheng^1*^

^1^College of Agronomy, Sichuan Agricultural University, Chengdu, China

^1^All authors have same affiliation

*Author to whom correspondence should be addressed:

Aiping Zheng, apzh0602@gmail.com

Aijun Wang and Li Ma have contributed equally to this work.

**Supplementary Figures**


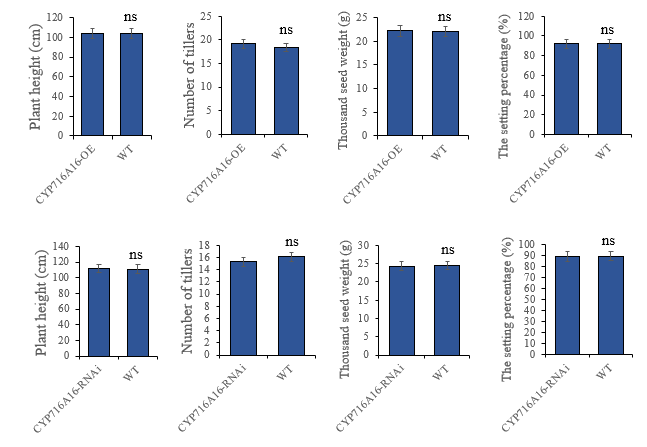


Supplementary Figure S1 The data of important agronomic traits of WT, CYP716A16-OE, CYP716A16-RNAi lines.

OsACS2

OsAOC


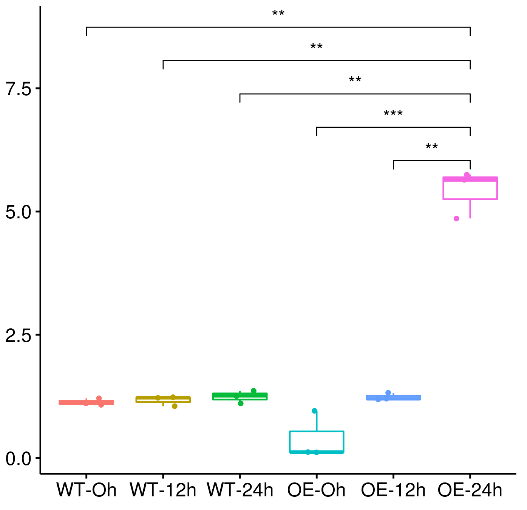

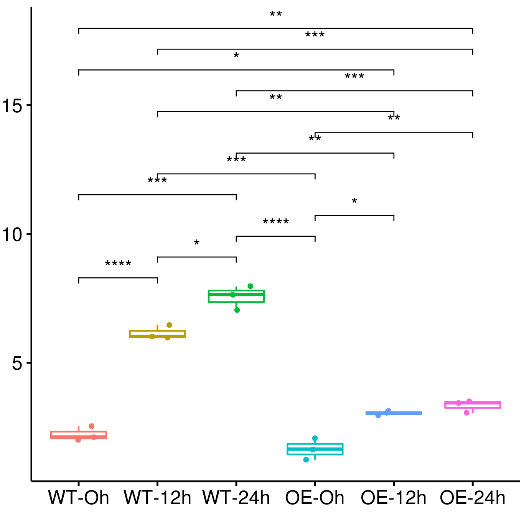


Relative expression

Relative expression

OsNPR1


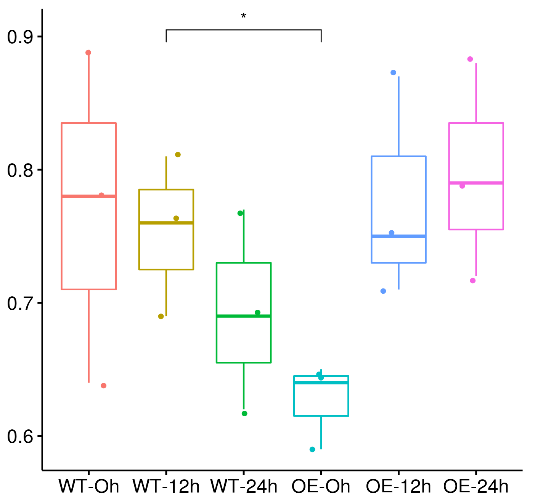


Relative expression

Supplementary Figure S2 The expression pattern of OsAOC, OsACS2, and OsNPR1 in WT and CYP716A16-OE plants after inoculation with *R. solani* AG1-IA.


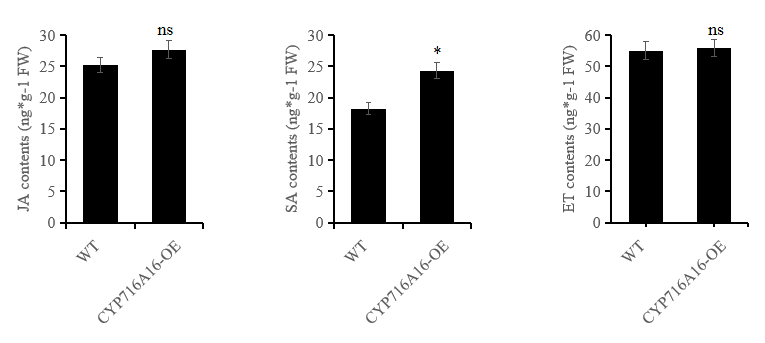


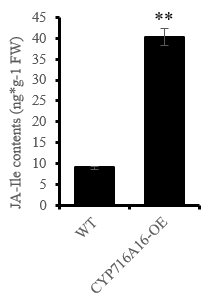


Supplementary Figure S3 The levels of (JA, JA-Ile, SA, and ET) in WT and CYP716A16-OE plants after inoculation 24 h with *R. solani* AG1-IA.


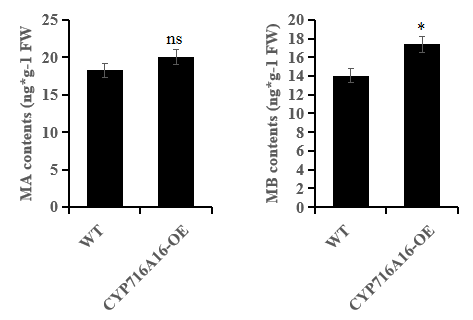


Supplementary Figure S4 The contents of phytoalexin (MA and MB) in WT and CYP716A16-OE plants after inoculation 24 h with *R. solani* AG1-IA.
